# Protective effect of bacterial lipase on lipopolysaccharide-induced toxicity in rat cardiomyocytes; H9C2 cell line

**DOI:** 10.34172/jcvtr.2020.06

**Published:** 2019-12-23

**Authors:** Mina Mamipour, Mohammadreza Yousefi, Alireza Dehnad, Yousef Faridvand, Reza Zarezadeh, Majid Khaksar, Ayda Pouyafar, Reza Rahbarghazi

**Affiliations:** ^1^Department of Biotechnology, Higher Education Institute of Rab-Rashid, Tabriz, Iran; ^2^Stem Cell Research Center, Tabriz University of Medical Sciences, Tabriz, Iran; ^3^Biotechnology Department, East Azerbaijan Research and Education Center Agricultural and Natural Resources, AREEO, Tabriz, Iran; ^4^Infectious and Tropical Diseases Research Center, Tabriz University of Medical Sciences, Tabriz, Iran; ^5^Department of Biochemistry and Clinical Laboratories, Faculty of Medicine, Tabriz University of Medical Sciences, Tabriz, Iran; ^6^Department of Applied Cell Sciences, Faculty of Advanced Medical Sciences, Tabriz University of Medical Sciences, Tabriz, Iran

**Keywords:** Rat Cardiomyocytes, Lipopolysaccharide, Lipase, Toll-like Receptor Signaling, Cell Cytotoxicity

## Abstract

*** Introduction:*** Cardiovascular system is highly sensitive to LPS-induced oxidative damage. This study aimed to show the inhibitory effect of bacterial Lipase on LPS-induced cardiomyoblasts toxicity.

*** Methods:*** Rat cardiomyoblasts H9C2 were classified into Control, LPS (cells received 0.1, 1 and 10 μg/mL LPS) and LPS+ Lipase groups. In LPS+Lipase group, different concentrations of lipopolysaccharide were pre-incubated with 5 mg/mL bacterial lipase at 37˚C overnight prior to cell treatment. After 72 hours, cell viability was assessed by MTT assay. The expression of key genes related to toll-like receptor signaling pathways was assessed by real-time PCR assay. Percentage of fatty acids was evaluated in each group using gas chromatography assay. The levels of NO was also measured using the Griess reaction.

*** Results:*** Data showed H9C2 cells viability was decreased after exposure to LPS in a dose-dependent manner (*P* < 0.05). Incubation of LPS with lipase increased cell survival rate and closed to near-to-control levels (*P* < 0.05). Lipase had the potential to blunt the increased expression of IRAK and NF-κB in cells after exposure to the LPS. Compared to the LPS group, lipase attenuated the increased level of NO-induced by LPS (*P* < 0.05). Gas chromatography analysis showed the reduction of saturated fatty acids in cells from LPS group while the activity of lipase prohibited impact of LPS on cell fatty acid composition. LPS decreased the ability of cardiomyoblasts to form colonies. Incubation of LPS with lipase enhanced clonogenic capacity.

***Conclusion:*** Reduction in lipopolysaccharide-induced cytotoxicity is possibly related to lipase activity and reduction of modified lipopolysaccharide with toll-like receptor.

## Introduction

Lipopolysaccharide (LPS) or endotoxin is one of the most important parts in the cell wall of gram-negative bacteria, which is responsible for the various organ toxicities, especially myocardial pathologies promoted by the induction of pro-inflammatory cytokines.^1,2^ LPS is produced by destroying bacteria exposed to antibiotics and complement system.^3^ Production of pro-inflammatory cytokines triggers multiple intracellular signaling pathways inside cardiomyocytes.^4,5^ According to results from different experiments, LPS could induce autophagic state and hamper the expression of different factors participating in dynamic cell proliferation and stability.^6^ Toll-like receptors (TLR family), which is formed of at least 10 mammalian homologs of Drosophila toll, function as pattern recognition receptors for bacterial LPS and thereby control the production of pro-inflammatory cytokines.^7,8^ A member of this family named TLR-4 functions as the main receptor for LPS of gram-negative bacteria, transduces signals through effector MyD88, interleukin-1 (IL-1) receptor-associated kinase (IRAK), and TRAF6 and then activates NF-ƙB and mitogen-activated protein kinase pathway.^9,10^ Some of TLRs like -1, -2, -4, and -6, unlike TLR-4, constitute heterodimers, while a unique homodimer has been described for TLR-4.^11^ Also, a protein named MD-2 is necessary for efficient LPS signal transduction, making a complex with the extracellular domain of TLR-4.^12-14^ It is also proposed that the attachment of soluble serum proteins such as CD14, LPS binding protein (LBP) to bacterial LPS enhances affinity of LPS to TLR-4.^2^ CD14 in membrane-bound form is also candidate as an alternative receptor for LPS and acts as enhancer for TLR signaling pathway.^2^ The endotoxic domain of bacterial LPS contains phosphorylated lipid A with affinity to TLR, inducing cytotoxicity in target cells.^11^ Lipid A is phosphorylated by two phosphates and fatty-acylated by a different number of fatty acids.^11^ The number of fatty acids and phosphate groups is important in the recognition of LPS by TLR4 and MD-2 complex.^15^ Modification of lipid A structure can alter the immune response and signaling pathways.^15,16^ Bacterial lipases are a group of enzymes that could hydrolyze triglyceride to fatty acids and secreted by different bacteria to surrounding condition.^17^ Here, we hypothesize that enzymes belong to the class of lipase could blunt the detrimental effects of LPS by hydrolyzing and molecular structure. To the best of our knowledge, there are no previous studies examining the possible inhibitory effects of lipase on bacterial LPS and its interaction with cell membrane receptors. The current experiment is a preliminary attempt to address this issue. In the present study, we have investigated the neutralizing effects of bacterial lipase on LPS activity transduced via TLR signaling in rat cardiomyocyte cell line H9C2. The results of the current experiment could shed light on the potency of lipase in the LPS biological activity in cardiovascular system.

## Materials and Methods

### Cell culture

In the current experiment, rat cardiomyoblasts H9C2 cells were obtained from Pasteur Institute (Tehran, Iran). Cells were plated in T25 cm² flasks in high-glucose content Dulbecco’s modified Eagle’s medium (DMEM, Gibco) supplemented with 10% fetal bovine serum (FBS), and 1% Pen-Strep (Gibco). The cells were maintained under a humidified atmosphere with 5% CO_2_ at 37˚C.^18^ For subculturing, 0.25% Trypsin-EDTA (Gibco) solution was used. The cells at passage 3 were subjected to various analyses.

### LPS and a lipase treatment

To detect the possible neutralizing activity of bacterium lipase on LPS structure, we aimed to examine *Pseudomonascepasia-*derived lipase (Cat no: 62309, Sigma) on LPS before adding LPS to the culture medium. In the current experiment, cells were classified into three main groups as follows; Control, LPS, and LPS plus lipase groups. No treatment was added to the control cells. In LPS group, cells were treated with different concentrations of LPS including 0.1, 1 and 10 µg/mL for 72 hours. In the LPS + Lipase group, cells received the same concentration of LPS which were incubated with 5 mg/ml lipase at 37˚C overnight. Prior to adding to the culture medium, the mixture of LPS and lipase was exposed to 56˚C for 20 minutes in order to inhibit enzyme activity.

### MTT assay

To detect LPS IC_50_ value in rat myocardioblastoma, different concentrations of *Klebsiella pneumonia*-derivedLPS (Cat no: L4268, Sigma), including 0.1, 1, and 10 µg/mL, were used.^19^ An initial cell density of 1 × 10^4^ cells was plated in each well of 96-well plates (Surfa) and cells allowed to reach appropriate confluency (70-80%). We added 200 µL culture medium supplemented with 1% LPS and different doses of LPS into each well. Cell survival rate was determined by MTT (3-(4, 5-Dimethylthiazol-2-yl)-2, 5-diphenyltetrazolium bromide) assay with some modifications.^20,21^ After 72 hours, the supernatant was removed and 100 μL MTT solution (dilution: 1 mg/mL, Sigma) transferred into each well. Then, plates were kept at 37˚C for 4 hours and 50 μL dimethyl sulfoxide (Merck) was added to each well. Optical density was measured at 570 nm using a microplate reader (Model: ELX808, BioTek). The percentage of cell viability was expressed as % of non-treated control cells. In the current experiment, three different sets of experiments were performed.

### Real-time PCR

The activity of bacterial LPS on TLR signaling was monitored following exposure to lipase. Here, we investigated the inhibitory effect of lipase on LPS-derived activity of genes MyD88, IRAK-1 and NF-κB. In brief, medium containing 1 × 10^6^ cells and 10% FBS were plated in each well of 6-well plates and maintained for 24 hours. Following reaching 70%-80% confluency, cells were classified into different groups as above-mentioned. The real-time PCR assay was performed according to the previous experiment.^22^ Total RNA was extracted by RNase extraction kit (Yekta Tajhiz Azma, Iran) and reverse-transcripted into cDNA using cDNA synthestas kit (Yekta Tajhiz Azma, Iran) according to the manufacturer’s protocol.^23^ The forward and reverse primers were designed by Oligo7 Primer Analysis software (Molecular Biology Insights, Inc.).^24^ The transcription of genes was determined using Corbbet (Rotor-Gene 2000) and SYBR green assay. The sequence of primers was outlined in Table 1.

**Table 1:** Primer list

**Gene**	**Forward**	**Reverse**	**Annealing Temperature (Ta)**
MyD88	GAGAAAAGGTGTCGTCGCAT	TTCTGTTGGACACCTGGAGA	58°C
IRAK1	CACCACTACAATCTTCAGCCC	CAAAACCACCCTCTCCAATCC	61°C
NF-ƙB	GAGACCTGGAGCAAGCCATT	TGGGGGCACGGTTATCAAAA	58°C
β-actin	TGACAGGATGCAGAAGGAGA	TAGAGCCACCAATCCACACA	58°C

### Gas chromatography assay

In order to determine the possible effect of bacterial lipase on integrity of LPS lipid A, we used gas chromatography (GC) technique as previously described.^25^ This method has the potential to separate total lipid contents from liquid complex mixtures. First, we prepared 100 μL of samples from groups LPS alone, 0.1 µg/mL LPS + Lipase, 1 µg/mL LPS + Lipase and 10 µg/mL LPS + Lipase. Then, 2 mL of benzene/methanol (C_6_H_6_/CH_3_OH) solution (4:1) containing internal standard were added to each sample. The reaction was initiated for transesterification by the addition of 0.2 ml acetyl chloride (CH_3_COCl), and the culture tubes were gently shaken for 1 minute. The samples were then heated at 70˚C for 1 hour in a water bath and transferred to capillary columns followed by the addition of 6% K_2_CO_3_ and benzene solutions. Thereafter, tubes were centrifuged at 1500 rpm for 5 minutes. Finally, the benzene phase was collected by using a Pasteur pipet‌to culture tubes. Fatty acids analysis was done on a Buck Scientific 610 Gas Liquid Chromatograph. Any changes in values were studied based on retention times after comparison with the internal standard and control sample.

### Determining the intracellular content of reactive oxygen species (ROS)

In order to determine the generation of ROS in rat cardiomyocytes, 1 × 10^4^ cells were plated in 96-well plates and treated for 72 hours as the above-mentioned. Thereafter, the supernatant was discarded and medium containing 1 µM dichloro-dihydro-fluorescein diacetate (DCFH, C_24_H_16_Cl_2_O_7_; Sigma) added. Plates were incubated at 37˚C for 40 minuntes.^26^ Cells were washed twice with phosphate-buffered saline (PBS). Finally, the fluorescence intensity of each sample was measured at 475 nm by using a microplate reader (BioTeK, USA). The values in different groups were normalized to non-treated control and expressed as relative fluorescence intensity.

### Nitric oxide measurement

To determine the nitric oxide (NO) production in LPS-treated cells, we used the Griess assay according to previous experiments.^27^ An initial cell density of 1 × 10^4^cells was seeded in each well of 96-well plates and classified into groups mentioned before. Based on the previous protocols, 100 μL of supernatant was incubated with 20 μL of Griess (50 mg sulfanilamide [Cat no: S9251, Sigma] in 5 mL 5% phosphoric acid [Merck, 100573]) for 10 minutes at room temperature. Then, 20 μL Griess B solution (6.95 mg N-(1-naphthyl) ethylenediamine dihydrochloride [Cat no: N9125] in 5 mL distilled water) was added to each well.^28^ After 2 minutes, the absorbance was measured at 540 nm using a microplate reader. The total NO concentration was expressed as nM.

### Clonogenicassay

The ability of rat H9C2 myoblasts to form colonies is touted as the functional behavior of stem cells.^29^ For this propose, we performed clonogenic assay with some modifications.^30^ In brief, 1000 H9C2 cells were suspended in the mixture of 1:1 methylcellulose/gelatin and culture medium (2X) and transferred into each well of 6-well plates. The cells were cultured for a period of 14 to 21 days. The number of colonies was counted on 7 random fields (high power field = HPF).

### Statistical analysis

In this study, data were presented as mean ± SD. For statistical analysis, one-way ANOVA with a post hoc Tukey test was performed by using GraphPad Prism software ver. 5 (GraphPad Software Inc.). *P*value level was considered statistically significant below 0.05. In histograms, the significant differences between groups were shown by brackets and asterisks.

## Results

### Lipase decreased the detrimental effect of LPS on rat cardiomyocytes

**Figure 1:**
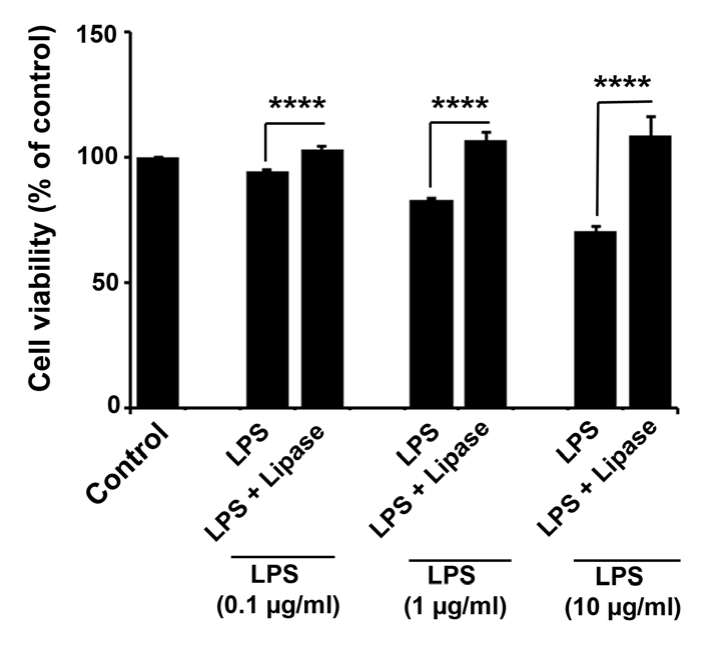
Cell treatment with 0.1, 1 and 10 μg/mL LPS for 72 h (**A**). MTT assay revealed a dose-dependent reduction in cell viability after exposure to 0.1, 1 and 10 μg/ml of LPS. Incubation of rat cardiomyocytes with the combination of LPS and Lipase for a period of 72 h (**B**). An enhanced cell survival rate was shown in LPS-treated cells after exposure to bacterial Lipase (5 mg/mL). This experiment was repeated in triplicate (each in octuplicate). Student *t*-test (for two groups) and one-way ANOVA with Tukey test (for more than two groups). ***P < 0.001; ****P < 0.0001. LPS: Lipopolysaccharide.

According to data from the MTT assay, we noted that LPS influenced cell viability in a dose-dependent manner (*P* < 0.05; Figure 1A). By increasing the concentration of LPS from 0.1 to 10 µG, the number of viable cells was reduced (*P*_Control vs. 0.1, 1 and 10 µg/mL LPS_ <0.001; *P*_0.1 µg/mL LPS vs. 1 µg/mL LPS_ <0.001 and *P*_ 1 µg/mL LPS vs. 10 µg/mL LPS_ <0.001). The data demonstrated that the cell viability in the group given 0.1 µg/mL LPS with 93% viability reached to 69.53 ± 3.15% in cells treated with 10 µg/mL LPS compared to the control group, showing dose-dependent toxicity of LPS on rat cells. Next, we analyzed the neutralizing effect of lipase on bacterial LPS. MTT assay indicated that lipase successfully blunted the cytotoxic effect of LPS on rat cardiomyocytes indicated by increased survival rate as compared to control-matched groups (*P*_Lipase vs. 0.1, 1 and 10 µg/mL LPS_ <0.0001; Figure 1B). These data showed the potency of bacterial-derived lipase on neutralizing the detrimental LPS effects on cardiac cells.

### Lipase treatment inhibited the activated TLR-4 signaling pathway induced by LPS

**Figure 2:**
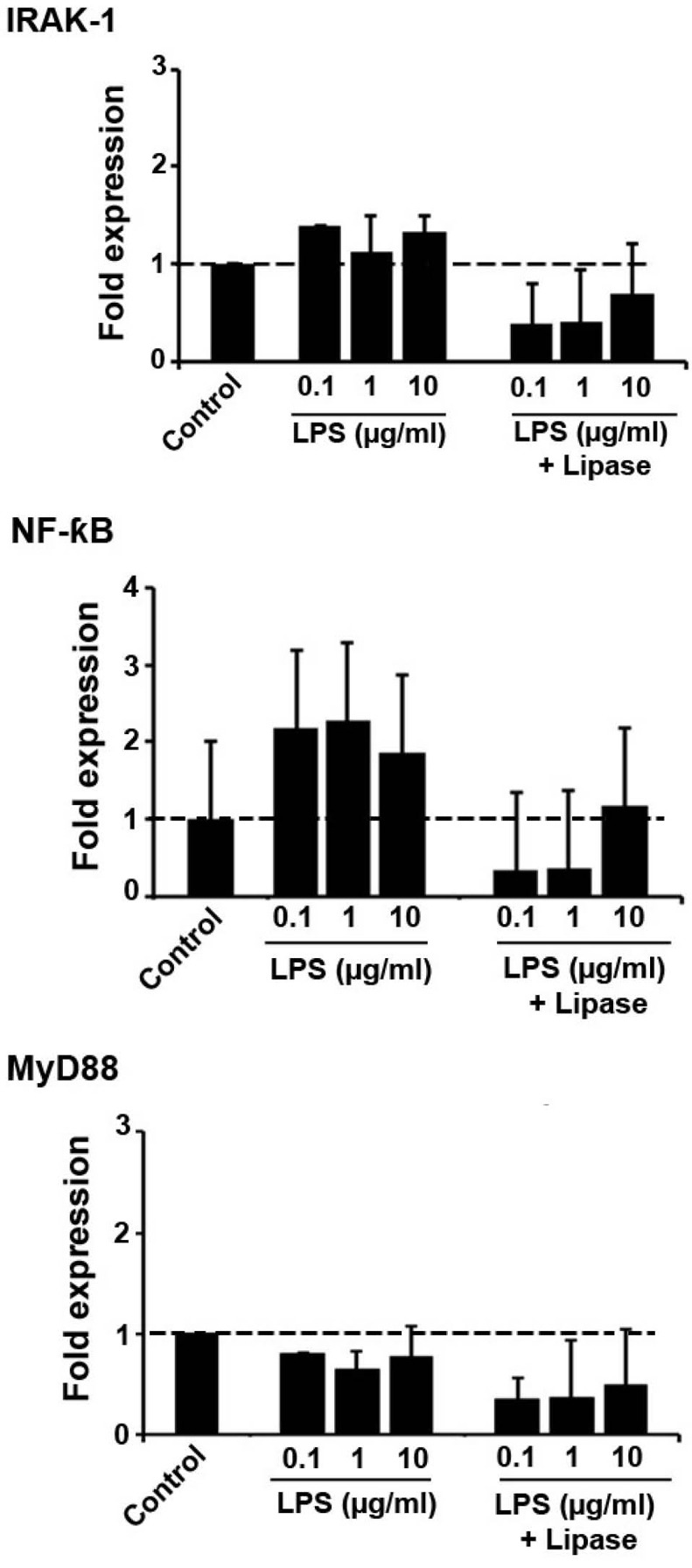
The real-time PCR analysis of TLR signaling in rat H9C2 cells following treatment with LPS and Lipase. Data showed that LPS had the potential to increase the transcription of IRAK-1 and NF-κB, showing the induction of the TLR signaling pathway. No difference in the level of MyD88 was obtained after treatment with LPS and Lipase (n=3). One-way ANOVA with Tukey post hoc test *P < 0.05; **P < 0.01 and ***P < 0.001. LPS: Lipopolysaccharide; IRAK: interleukin-1 (IL-1) receptor-associated kinase; MyD88: Myeloid differentiation primary response 88; NF-κB: nuclear factor kappa-light-chain-enhancer of activated B cells.

We performed real-time PCR analysis to monitor the dynamics of TLR-related signaling after the experimental procedure (Figure 2). We examined the expression of genes MyD88, IRAK-1 and cRel (a member of Nf-κB family) in H9C2 cells after exposure to the combined regime of lipase and LPS and LPS alone. Data analysis showed LPS potentially promoted the TLR signaling pathway by the modulation of MyD88, IRAK-1, and Nf-κB effectors. We found an increased transcription level of c-Rel (LPS_0.1 µg/mL_= 2.1-fold; LPS_1 µg/mL_= 2.2-fold and LPS_1 µg/mL_= 1.8-fold; (*P*_Control vs. 0.1 µg/mL LPS_ <0.001; *P*_Control vs. 1 µg/mL LPS_ <0.05 and *P*_Control vs. 10 µg/mL LPS_ <0.01) in all groups received LPS alone, showing the induction of c-Rel effector via TLR signaling. The level of IRAK-1 and MyD88 did not reach significant differences following LPS treatment alone or after enzymatic activity of lipase. The expression of IRAK-1 was also induced while a slight reduction in the expression of MyD88 observed (Figure 2). According to data from real-time PCR analysis, lipase blunted the increased TLR activity induced by LPS alone (Figure 2). Both transcription level of c-Rel and IRAK-1 seems to be decreased in cells given the combination of LPS and lipase. We found a 0.5-fold and 0.6-fold decrease in expression of IRAK-1 and cRel (Figure 2). Notably, the transcription of MyD88 was low in cells from LPS-treated and LPS + Lipase groups. These data possibly demonstrate that different pathways, unless MyD88, could activate the Nf-κB factor after TLR exposure to LPS. It is also possible to hypothesize that compensatory cell response could promote TLR signaling or an inhibitory feedback mechanism participates in the regulation of MyD88 through TLR signaling pathway.

### GC assay confirmed the molecular modification in LPS by lipase activity

**Figure 3:**
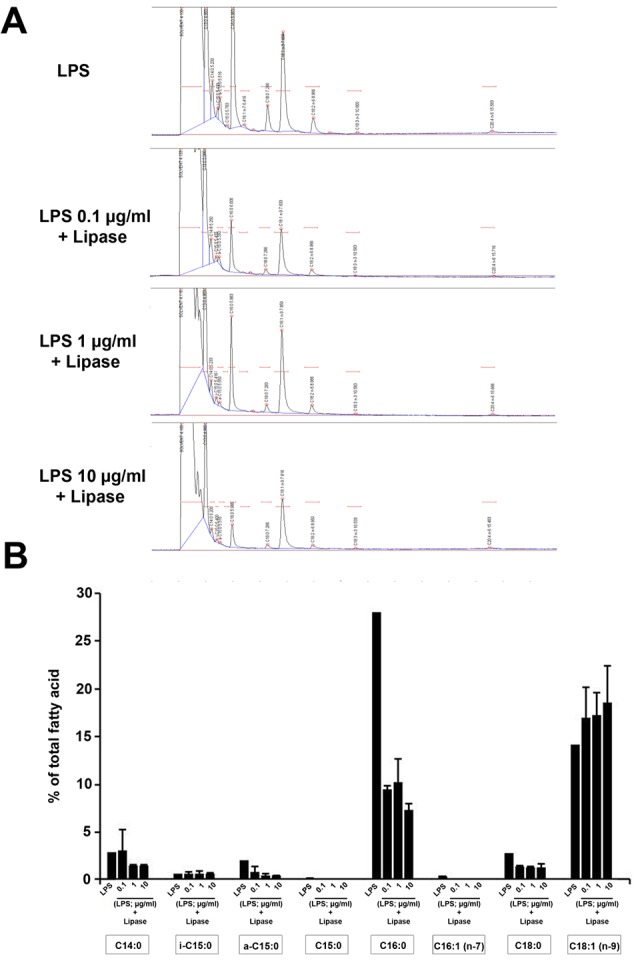
The GC analysis of total fatty acids in cell lysate after exposure to the combination of LPS and Lipase (A-B; *n* = 3). One-way ANOVA with Tukey test (for more than two groups). **P < 0.01 and ***P < 0.001. LPS: Lipopolysaccharide; C14:0 = Myristic acid; C15:0 = Pentadecylic acid; iC15:0 = isopentadecanoic acid; a-C15:0 = anteiso-Pentadecanoic acid; C16:0 = Palmitic acid; C16:1 (n-7) = Trans-palmitoleic acid; C18:0 = Stearic acid; and C18:1 (n-9) = Oleic acid.

To realize that lipase can affect LPS structure or not, we monitored the pattern of LPS fatty acid pre- and post-treatment with lipase. According to GC data, we showed the existence of saturated and unsaturated fatty acids such as C_14_, C_15_ (i-C_15:0_, a-C_15:0_, and C_15_ which are classified according to the location of CH_3_) C_16_, C_16:1_ and C_18_ (C_18_ and C_18:1_) (*P* < 0.05; Figure 3). Lipase treatment changed the percent of fatty acid composition in the structure of lipid A. Data showed a significant reduction in the amount of fatty acids a-C_15_ (*P*_Lipase vs. 0.1 µg/mL LPS_ <0.01 and *P*_Lipase vs. 1 and 10 µg/mL LPS_ <0.001), C_16_ (*P*_Lipase vs. 0.1, 1 and 10 µg/mL LPS_ <0.001) and C_18_ (*P*_Lipase vs. 0.1, 1 and 10 µg/mL LPS_ <0.001), indicating enzymatic activity of bacterial lipase on LPS composition and integrity. The level of fatty acids C_15:0_ and C_16:1_ was null in LPS-treated cells (Figure 3). These results could explain the blunting effect of lipase on LPS by the modulation of fatty acid composition in LPS lipid A.

### Lipase reduced intracellular ROS production induced by LPS

**Figure 4:**
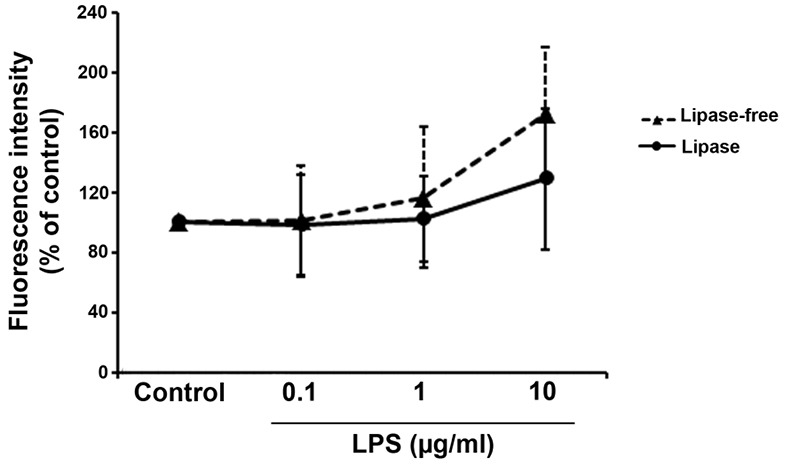
The inhibitory effect of Lipase in intracellular ROS content in LPS-treated cells. Based on our results, Lipase reduced increased ROS levels induced by LPS (n = 8). LPS: Lipopolysaccharide; ROS: reactive oxygen species.

According to the great body of literature, LPS has the potential to initiate the generation of ROS production via TLRs in different cell types like cardiomyocytes.^31^ In order to analyze the inhibitory effect of bacterial lipase to LPS-induced ROS production, we measured the content of ROS by DCFH-DA assay. By increasing the concentration of LPS, the ROS content was proportionally induced in which rat cardiomyocytes received 10 µg/mL LPS showed the highest ROS level (Figure 4). Despite these conditions, the differences between cells given LPS only or the combination of lipase and LPS did not reach significant levels (*P*>0.05). We noted a 45% increase in ROS production in cells treated with 10 µg/mL LPS in comparison with non-treated control. Noteworthy, data demonstrated that LPS treatment with lipase could impede the pro-inflammatory status, contributing to decreased ROS production (Figure 4). Compared to LPS-treated cells, the incubation of LPS with lipase caused a 17% reduction of ROS generation in cells from 10 µg/mL LPS (Figure 4). These features showed the inhibitory effect of lipase on LPS-induced ROS generation possibly governed by TLRs. In the current study, lipase had no detrimental effect on rat cardiomyocytes (Data not shown).

### Lipase could decrease the LPS-induced NO production in rat cardiomyocytes

**Figure 5:**
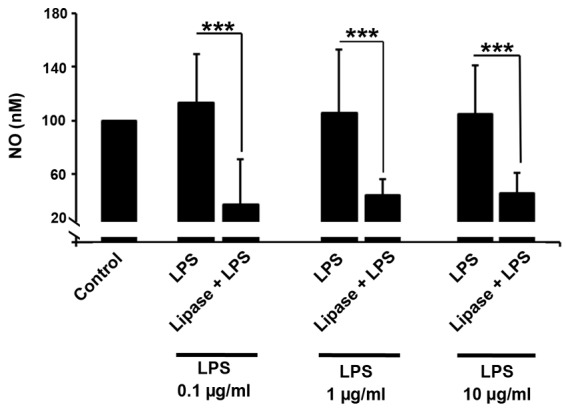
Detection of NO production in rat cardiomyocytes exposed to the combined regime of LPS and Lipase. Lipase decreased the nitrosative stress in rat cardiomyocytes as compared to the control and LPS-treated groups. This experiment was repeated in triplicate (each in octuplicate). Student t-test. ***P < 0.001. LPS: Lipopolysaccharide; NO: nitric oxide.

Our next effort was to determine the lipase activity on nitrosative stress-induced upon the treatment with LPS. Griess analysis confirmed an inhibitory effect of lipase on NO production in rat cardiomyocytes (Figure 5). Compared to the control cells, we found that the intracellular content of NO was increased in cells received 0.1, 1 and 10 µg/mL LPS but these changes did not reach statistically significant levels (*P>*0.05). Incubation of different LPS doses with lipase decreased the generation of NO and reached the levels below control (*P*_Control vs. 0.1 µg/mL LPS + Lipase_ <0.001; *P*_Control vs. 1 µg/mL LPS + Lipase_ <0.001_;_*P*_Control vs. 10 µg/mL LPS + Lipase_ <0.001). Similar to data from the ROS panel, it is reasonable to suggest lipase could inhibit ROS and nitrosative stress in cells being exposed to bacterial LPS.

### Lipase improved the reduced colony number induced by LPS

**Figure 6:**
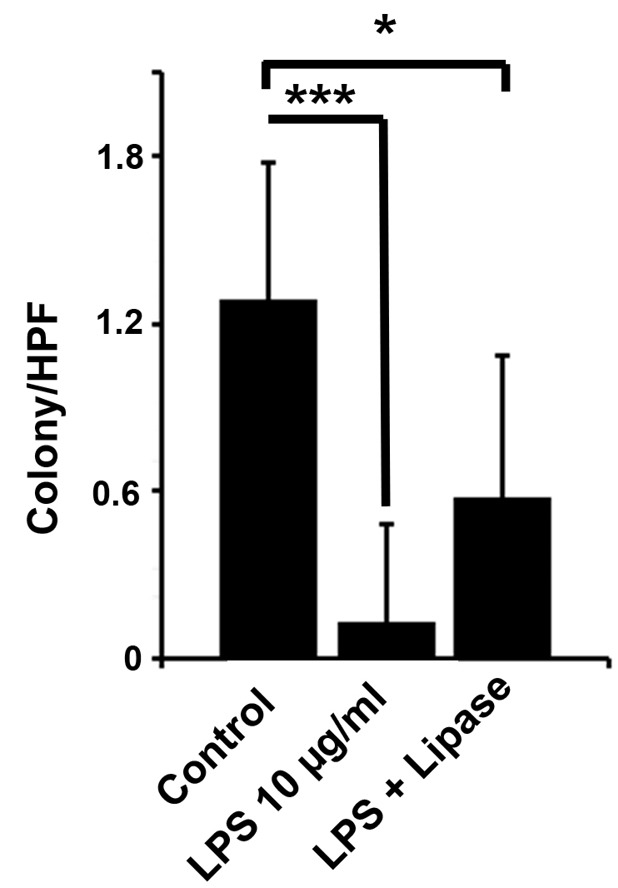
Effect of Lipase and LPS on the clonogenic capacity of rat H9C2 cells. As shown, Lipase increased the LPS-induced reduction of colony number in rat cells. One-Way ANOVA and Tukey post hoc analysis (n=3). *P < 0.05 and ***P < 0.001. LPS: Lipopolysaccharide; HPF = High power field.

To show whether lipase can affect the clonogenic property of rat cardiomyocytes in the presence of LPS, the colony formation assay was used. Micrograph observation showed the reduction of colonies in the LPS group compared to the control cells (Figure 6). The exposure of cells to LPS plus lipase contributed to an increased colony number (*P*_control 10 µg/mL LPS_ < 0.001; *P*_control versus LPS + lipase_ < 0.05). Considering data from this panel, LPS could inhibit the clonogenic activity of rat H9C2 cells after 72 hours. The changes in LPS composition via the application of lipase could return the ability of these cells to form colonies in vitro.

## Discussion

The present study outlines the protective effects of bacterial lipase on LPS-induced cardiotoxicity in rat H9C2 cell line. Based on clinical observations, cardiotoxicity is caused by sepsis shock with a high mortality rate of 40% up to 60% and there is no effective therapy found yet.^32^ Therefore, many attempts have been collected to find alternative therapy or prevent sepsis-related pathologies. We, here, found that LPS had the potential to induce cell toxicity after 72 hours while the combined regime of LPS and lipase yielded in near to normal cell survival rate. In our previous work, the detrimental effect of LPS was approved on different human intestinal cell lines such as HT-29, HCT-116, and SW48.^33^ It was previously mentioned that the toxicity of LPS depends on lipid A part, which includes fatty acids and two phosphate groups.^34,35^ Bentala et al detoxified LPS by removing one phosphate group from lipid A caused to formation of monophosphoryl lipid A (MPLA).^6,36,37^ We also found that detrimental effect of LPS was reduced after exposure to lipase, resulting in an improved cell survival rate. Commensurate with these statements, the intracellular level of ROS was similarly diminished upon treatment with the combined regime of lipase and LPS. Consistent with our data, Bentala and co-workers showed the reduction in intracellular ROS by dephosphorylation and thereby the complication of sepsis attenuated.^6^ It is thought that an increased content of ROS coincided with cytotoxicity in rat H9C2 cells.^38^ This result possibly correlate with the destruction of LPS structure and inability to attach TLR. These data showed that reduced cell survival rate in LPS-treated cells could relate to the promotion of oxidative and nitrosative stresses after TLR stimulation. In this study, we showed that lipase could decrease these effects by decomposing the LPS structure. Over activity of TLRs via bacterial LPS could exert detrimental effects on rat cardiac cells via the modulation of intracellular NO and ROS contents. Reduction in the levels of NO and ROS stands for a fact that incubation of LPS with bacterial lipase could change LPS backbone and decrease LPS-TLR interaction.

GC analysis showed a profound change in the molecular structure of LPS following the treatment with lipase. Noteworthy, we found a remarkable reduction in the level of saturated fatty acid in LPS backbone upon exposure to lipase. It seems that lipase catalyzes and initiates the biochemical reaction and reduces the levels of saturated fatty acids in the lipid A part of LPS.^39^ According to a plethora of experiments, lipid A compound is composed of both saturated and unsaturated fatty acids and biological activity of LPS depends on the existence of saturated fatty acids.^40^ These features support the neutralizing effects of lipase on LPS. Moreover, data from real-time PCR analysis indicated the up-regulation of both genes IRAK-1 and c-Rel, effectors of TLRs signaling pathway after cell exposure to LPS. We showed the reduction of both IRAK-1 and c-Rel genes, but not MyD88, in cell-being exposed to the combination of LPS with lipase. It was previously reported *Klebsiella pneumonia*-derivedLPS engaged different adaptor protein, termed MAL, rather than MyD88.^41^ Effector MAL participates in the signaling pathway of both TLR-2 and TLR-4.^42^ Noteworthy, some authorities declare MyD88 does not have a direct association with the TLR-4 system due to electro-positive charge on its surface, resulting in the weak interaction of these proteins with each other. In contrast, the existence of electro-negative charge on Mal protein enables easily interact with TLR-4.^43^ In line with these statements, one could hypothesize that the detrimental effect of LPS on rat cardiomyocytes is driven by TLR system by engaging effector rather than MyD88. We demonstrate the adverse effect of LPS on cellular behavior and molecular kinetics of H9C2 over the period of 72 hours. The protective effects of bacterial lipase were shown on LPS-induced cardiotoxicity in rat H9C2 cell line. Consistent with these descriptions, it is reasonable to hypothesize cardiovascular complications following sepsis could be managed by modulating TLR signaling pathway and control the interaction of gram-negative bacteria LPS with cell surface receptors. It seems that fatty acid removal from LPS structure could be touted as an appropriate strategy to inhibit the promotion of sepsis and cardiovascular insufficiency. Changes in LPS structure reduce the interaction of LPS with TLR and prohibit the induction of intracellular effectors. More investigation is needed to elucidate the underlying mechanism related to blunting effect of bacterial lipase on LPS.

There are some limitations related to the current experiment. For example, it is unclear to what extent other TLRs are stimulated by using bacterial LPS. The use of TLRs inhibitor could help us to precisely generalize the changes in the activity of downstream effectors to bacterial LPS. We also are not sure that to what extent LPS-degradable products are able to activate and/or inhibit the TLRs.

## Competing interests

Authors declare there is no potential conflict of interest.

## Ethical approval

Not applicable.

## Funding

This study was supported by a grant (IR.TBZMED.REC.1397.998) from Tabriz University of Medical Sciences.
